# The E3 ubiquitin ligase MAEA promotes macrophage phagocytosis and inhibits gastrointestinal cancer progression by mediating PARP1 ubiquitination and degradation

**DOI:** 10.7150/ijbs.102796

**Published:** 2025-02-10

**Authors:** Yanchun Feng, Xiangcai Zou, Jintuan Huang, Zhenze Huang, Guanghao Kuang, Yingming Jiang

**Affiliations:** 1 Department of Thyroid and Hernia Surgery, The Second Affiliated Hospital, Guangzhou Medical University, Guangzhou 511436, Guangdong, China.; 2 Microbiome Medicine Center, Department of Laboratory Medicine, Zhujiang Hospital, Southern Medical University, Guangzhou, Guangdong, China.; 3 Department of General Surgery (Department of Gastric Surgery Section 2), The Sixth Affiliated Hospital, Sun Yat-Sen University, Guangzhou 510655, Guangdong, China.; 4 General Surgery Center, Department of Thyroid Surgery, Zhujiang Hospital, Southern Medical University, Guangzhou, Guangdong, China.

**Keywords:** MAEA, Gastrointestinal cancer, PARP1, Macrophage, Phagocytosis

## Abstract

**Background:** While a role for the E3 ubiquitin ligase MAEA (macrophage erythroblast attacher) has been reported in several cancer types, its importance and mechanistic functions in gastrointestinal cancer (GIC) have yet to be established.

**Methods:** The functions of MAEA in GIC were explored through *in vitro* and *in vivo* experiments, including loss- and gain-of-function analyses. Mass spectrometry was used to identify proteins that interact with MAEA. The mechanisms through which MAEA influences tumor aggression were examined through immunoprecipitation analyses.

**Results:** GIC patients exhibiting reduced expression of MAEA were found to exhibit worse disease-free and overall survival outcomes. MAEA was found to impair the proliferation and chemoresistance of GIC tumors *in vitro* and in subcutaneous xenograft model systems. The combination of MAEA and the PARP1 inhibitor veliparib resulted in enhanced oxaliplatin treatment efficacy *in vivo.* From a mechanistic perspective, MAEA was found to mediate the K48-linked ubiquitination and degradation of PARP1, in addition to suppressing the M2 polarization of macrophages and enhancing macrophage phagocytic activity.

**Conclusions:** These data suggest that MAEA offers value as a prognostic biomarker and target for the treatment of GIC owing to its ability to degrade PARP1 and augment the phagocytic activity of macrophages.

## Introduction

Gastrointestinal cancers (GIC), including both gastric cancer (GC) and colorectal cancer (CRC) are characterized by a high degree of morbidity and poor patient outcomes 1, 2. Even with the advent of novel screening and treatment strategies, the effective treatment of these cancers remains very difficult 2, 3. Chemotherapy is among the most common approaches to GIC treatment 4, enabling the preferential targeting of rapidly dividing tumor cells 5, 6. Chemotherapeutic drugs can be applied alone or together with surgical, radiotherapeutic, and/or immunotherapeutic interventions. Innate or acquired chemoresistance, however, remains a persistent barrier to efficacious chemotherapeutic treatment, enabling a subset of tumor cells to resist the cytotoxic effects of these drugs. The presence of these drug-resistant cells ultimately leads to treatment failure, recurrent disease, and a poor prognosis. Several mechanisms can give rise to chemoresistance, including decreases in the rate of drug uptake, the enhancement of drug efflux, changes in drug targets, the enhancement of DNA damage repair, survival pathway activation, and changes in the microenvironmental conditions within and around the tumor 6. Efforts to overcome such chemoresistance represent an important strategy for oncology care in the clinic, underscoring the need for innovative therapeutic approaches including targeted drugs, immunotherapies, combination treatment regimens, and personalized medicine 7. The effective elucidation of the molecular mechanisms that underlie chemoresistance and the establishment of biomarkers that can reliably predict patient treatment responses are also vital steps toward the ability to improve patient outcomes and to prolong survival 8.

Poly ADP-ribose Polymerase 1 (PARP1) is a key enzyme involved in the DNA repair pathway that is a central regulator of cellular responses to chemotherapeutic drug exposure 9. The complex interactions between PARP1 and chemoresistance are closely tied to treatment outcomes 10. DNA damage induced by chemotherapy leads to the activation of PARP1, thereby triggering DNA repair 11, ultimately enabling tumor cells to survive and thereby contributing to chemoresistance. Efforts to target PARP1 have thus attracted significant attention as a means of overcoming chemoresistance and improving the efficacy of established chemotherapy regimens 12, 13. When combined with chemotherapy, PARP inhibitors capable of suppressing the activity of PARP1 provide clinical benefits, particularly when treating tumors with deficiencies in the DNA repair pathway such as those harboring BRCA mutations 13-15. The detailed characterization of interactions between PARP1, chemotherapeutic treatment, and chemoresistance is essential in order to guide the design of treatment strategies that are more effective, thus providing cancer patients with a better prognostic outlook.

The E3 ubiquitin ligase MAEA (macrophage erythroblast attacher) has been shown to preserve the function of hematopoietic stem cells through its ability to restrict cytokine receptor signaling activity in an autophagy-dependent manner 16. In a previous report, our team found that MAEA is also capable of degrading vimentin and suppressing cellular proliferation 17. Another recent report demonstrated that MAEA can facilitate PHD3 ubiquitin-mediated degradation, ultimately favoring glioblastoma progression through enhanced stemness and the augmentation of temozolomide resistance 18. While these studies highlight the close relationship between MAEA activity, stemness, and chemoresistance, no studies of the functional role of MAEA in GICs have been performed to date, highlighting a need to examine its importance in GC and CRC cases.

Here, a significant decrease in MAEA expression was observed in GC and CRC tissues as compared to healthy control tissues. The prognosis of patients expressing higher MAEA levels was more favorable than that of patients expressing lower levels of this E3 ligase. From a mechanistic perspective, MAEA was found to promote the K48-linked ubiquitination and degradation of PARP1, in addition to suppressing M2 macrophage polarization and enhancing the phagocytic activity of macrophages. The synergistic benefits of these effects may explain the observed inhibition of cellular proliferation and oxaliplatin resistance associated with MAEA.

## Materials and methods

### Patient samples

In total, 168 primary tumor tissue samples from Sun Yat-Sen University, Sixth Affiliated Hospital in Guangzhou, China were acquired from December 2007 - March 2012, as reported previously 17, 19, 20. These samples and the corresponding clinicopathological data were used to guide the construction of tumor microarrays used for immunohistochemical (IHC) staining.

### Immunohistochemistry

IHC staining was conducted using a biotin-streptavidin HRP conjugation approach (ZSGB Bio, China) as in prior reports 19. ThAntibodeis used for this study were specific for MAEA (28363-1-AP, Proteintech, China, 1:400), Ki67 (#9449, Cell Signaling Technology, China, 1:400), PARP1 (#9532, Cell Signaling Technology, 1:200) and Cleaved Capase-3 (#9664, Cell Signaling Technology, 1:2000).

### Cell culture

The THP1, NUGC3, HCT116, DLD1, and MKN45 cell lines from the Type Culture Collection Cell Bank of the Chinese Academy of Sciences Committee (Shanghai, China) were cultured in RPMI-1640 (Corning, USA) with 10% FBS (Gibco, USA) in a humidified 37°C 5% CO_2_ incubator. THP1 cell differentiation into macrophages was performed using an established approach 21.

### Migration analyses

After transfection and macrophage differentiation, THP1 cells were resuspended in serum-free DMEM and added to the upper portion of a Transwell chamber, with the lower chamber being filled with DMEM containing 10% FBS. Following a 24 h incubation, cells were fixed, stained, and analyzed as in past reports 19, 21.

### Transfection

A PCR amplicon was generated from the full-length MAEA open reading frame (ORF; NM_001017405.3), after which it was inserted into the HA-tagged pCDH-CMV-MCS-EF1-CopGFP-T2A-Puro (PCDH) lentiviral vector. Separately, MAEA (NM_001017405.3) and PARP1 (NM_001618.4) plasmids were introduced into the pCDNA3.1 vector containing a MYC, Flag, or His tag. The HA-tagged Ub-K63 and Ub-K48 plasmids were obtained from Addgene. MAEA-specific shRNAs (shRNA-1, 5-GCAAGAAAGCACTTCAGCCAA-3; shRNA-2, 5-CCCGAGAACCAAAGAAGTCTT-3) were from Genepharma (Shanghai, China). Stable cell lines were generated through lentiviral transduction, with transient infection having been performed as reported previously 19.

### qPCR

RNA was extracted with an appropriate kit (EZB-RN4, EZBioscience, China), and another kit (EZB-RT2GQ, EZBioscience) was used for cDNA preparation, after which qPCR was performed as in past reports 23, 24.

Utilized primers included the following: GAPDH, 5-GACAGTCAGCCGCATCTTCTT-3 (forward) and 5-AATCCGTTGACTCCGACCTTC-3 (reverse); MAEA, 5-GAGACTGGACGCTGTGAGAC-3 (forward) and 5-AGGTCCTTGTACGGGGAGATG-3 (reverse); PARP1, 5-GTGGTCGGGACTGTCTCTAAG-3 (forward) and 5-TCTCCAGTAGCAACCTGAAAAGT-3 (reverse); CD206, 5-TCCGGGTGCTGTTTCCCTA-3 (forward) and 5-CCAGTCTGTTTTTGATGGCACT-3 (reverse); ARG1, 5-GTGGAAACTTGCATGGACAAC-3 (forward) and 5-AATCCTGGCACATCGGGAATC-3 (reverse).

### Western immunoblotting

RIPA buffer (Service-Bio, Wuhan, China) containing protease/phosphatase inhibitors was used to extract cellular protein, after which a BCA kit (Service-Bio) was used to quantify protein levels. Western immunoblotting was then performed as reported previously, using antibodies (all diluted 1:1000) specific for GAPDH (60004-1-Ig, Proteintech, China), MAEA (28363-1-AP, Proteintech), HA-tag (66006-2-Ig, Proteintech), Flag (F1804, Sigma, China), 6×His (10001-0-AP, Proteintech), and Myc-tag (60003-2-Ig, Proteintech), PARP1 (#9532, Cell Signaling Technology) and cleaved capase-3 (#9664, Cell Signaling Technology).

### Apoptosis assays

As in our previous study 20, cells were seeded in 6-well plates at a density of 2×10^5^ cells per well and incubated for 48 hours with or without OXA. Apoptotic cell death was analyzed by staining the cells with an Annexin V-APC/7-AAD kit (MultiSciences, Hangzhou, China) according to the manufacturer's instructions. The stained cells were then examined using a flow cytometer (Beckman Coulter, CA, USA), and the data were processed using FlowJo v10.0 (BD Biosciences, OR, USA) or CytExpert v2.4 (Beckman Coulter, CA, USA).

### Colony formation assays

After seeding cells in a 6-well plate (2x10^4^/well) for 24 h, cells were treated with or without oxaliplatin (OXA, TargetMol, Shanghai, China). They were then cultured for 10-14 days, fixed using 4% paraformaldehyde, stained using crystal violet, and imaged via microscopy. Images were captured with an Olympus camera (Tokyo, Japan) and processed in ImageJ. In 3D colony formation assays, 2000 cells in 200 μL of culture medium were added to an ultra-low attachment microplate (7007; Corning, USA) followed by culture for 12 days, changing media every 3 days. Cells were treated with appropriate combinations of Veliparib and/or oxaliplatin. An Incucyte Zoom system was used to image tumor spheres every 4 h, enabling the calculation of their volumes (Volume = 4/3πR^3^).

### Proliferation assays

After incubating cells in 06-well plates (2x10^3^/well) for 24 h, media was exchanged for media that was unsupplemented or contained Veliparib and/or oxaliplatin. Cells were imaged every 2-4 h with an Incucyte Zoom system, and viability was analyzed with a CCK-8 kit.

### Co-immunoprecipitation

After extracting proteins using a low salt lysis buffer, lysates were centrifuged at 4°C and the supernatants were combined with appropriate antibodies and beads (HY-K0202-5mL, MCE, USA) overnight at 4°C, after which all experiments were performed as in our past report 17, 19. The utilized antibodies (all diluted 1:1000) were specific for HA-tag (66006-2-Ig, Proteintech), Flag (F1804, Sigma), 6×His (10001-0-AP, Proteintech), and MYC-tag (60003-2-Ig, Proteintech).

### Mice experiment

BALB/c nude mice from GEMPHARMATECH (Guangdong, China) were housed in the Experimental Animal Center of the Sixth Affiliated Hospital at Sun Yat-sen University. Five female BALB/c nude mice (4 weeks old) were subcutaneously implanted in the left flank with 5 x 10^6^ tumor cells in 100 ul of PBS. Tumor volume was measured every 3 days (V = W^2^ * L/2). After 4 weeks, mice were euthanized and tumor size was measured.

For GC patient-derived xenograft (PDX) experiments, NOD-SCID mice from GEMPHARMATECH (Guangdong, China) were used. GC tissues from patients used in this study were collected and used with approval from Sun Yat-Sen University (SYSU-IACUC-2022051303) and patient approval. PDX mice were established as in prior studies 17, 22, and were randomized into four groups (n=5/group): Vector lentivirus, MAEA lentivirus, MAEA lentivirus+OXA, or MAEA lentivirus+OXA+Veliparib groups. Every three days, these mice were injected intraperitoneally with OXA (10 mg/kg). After 3 weeks when the tumors were 50-100 mm^3^ in size, the mice were euthanized. The tumors were harvested, fixed with formalin, paraffin-embedded, and used for IHC analyses.

### Database analyses

Correlations between the mRNA levels of MAEA and the DFS or OS of GIC patients were analyzed with the Kaplan-Meier Plotter database (https://kmplot.com/analysis/index.php?p=background), accessed on 12 June 2023.

### Statistical analyses

GraphPad Prism 7.0 (CA, USA) and SPSS 21.0 (IBM, NY, USA) were used for data analyses. Student's t-tests were used to analyze continuous data, which are reported as means and standard deviations. Categorical variables were analyzed with chi-square or Wilcoxon signed-rank tests as appropriate. Kaplan-Meier curves and log-rank tests were employed when analyzing survival. Multivariate analyses of prognostic factors were conducted using a Cox proportional hazards regression model with the backward-elimination method.

## Results

### High levels of MAEA expression are correlated with improved GC and CRC patient outcomes

**Figure 1:**
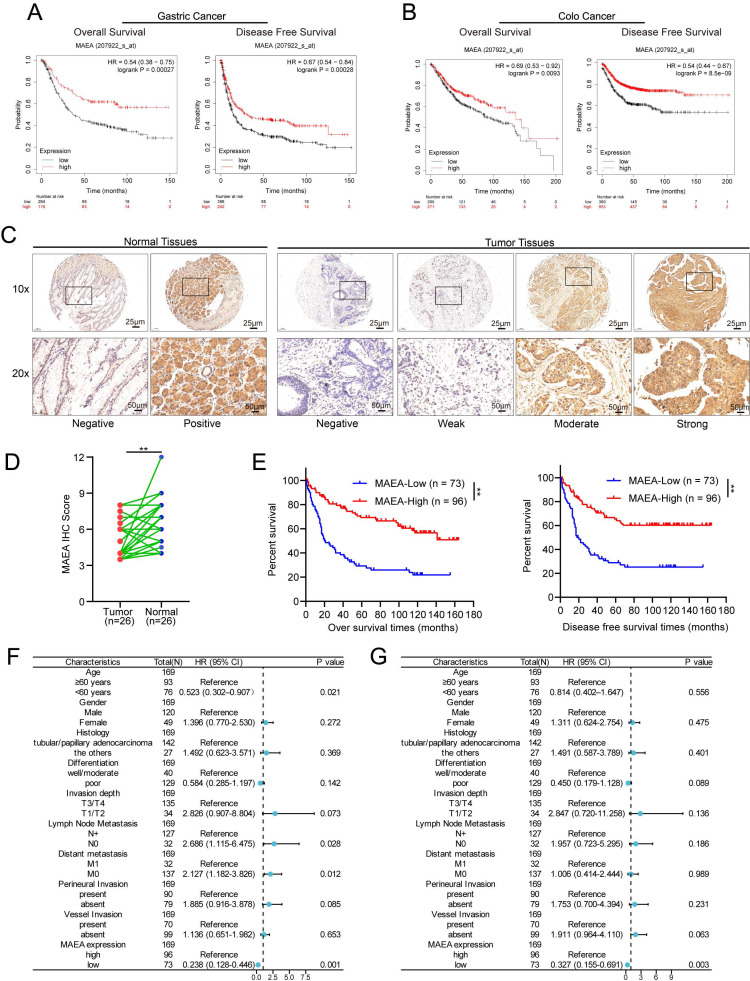
** High MAEA levels are correlated with improved GC and CRC patient outcomes. A-B** Kaplan-Meier Plotter analyses revealed a significant association between high MAEA mRNA levels and better DFS and OS for GC and CRC patients. **C** Representative images of MAEA expression in GC and paracancerous tissues. **D** Comparison of mAEA protein levels in 26 paired GC and paracancerous tissues. **E** OS and DFS as a function of MAEA expression was analyzed in GC patients. **G-H** Forest plots representing the outcomes from multivariate Cox regression analyses showing the prognostic performance of different variables as predictors of GC patient OS and DFS. *p < 0.05, **p < 0.01, ***p < 0.001.

Analyses of data from the Kaplan-Meier Plotter database indicated that high levels of MAEA expression in GC patients were associated with significantly better overall and disease-free survival (OS and DFS) relative to low MAEA levels (Fig. 1A), and the same was also true in CRC patients (Fig. 1B). Relative to paired paracancerous tissue samples, significantly lower levels of MAEA expression were evident in GC tumors from 26 patients (Fig. 1D). Consistently, IHC analyses of 169 GC tumor tissue samples revealed that the OS and DFS of patients with high MAEA levels were significantly better than those of patients with low levels of MAEA expression (Fig. 1E). When the relationship between MAEA protein levels and various clinicopathological characteristics was examined, low MAEA levels were found to be associated with worse Lauren's classification, distant metastasis, and perineural invasion (Supplementary Table 1). In multivariate regression analyses, MAEA expression was established as an independent predictor of both OS and DFS (Fig. 1F, G). These results thus revealed an association between lower MAEA levels and worse survival outcomes in patients with GC and CRC.

### MAEA is related to GC and CRC patient clinicopathological characteristics and survival outcomes

Using IHC data, correlations between the clinicopathological characteristics of 169 GC patients and MAEA expression levels were analyzed. Similarly, the correlative relationship between MAEA levels and clinicopathological characteristics were evaluated for 644 CRC patients in the TCGA database. These analyses revealed that low levels of MAEA expression in GC were associated with significantly higher rates of distant metastasis and perineural invasion, as well as worse Lauren's classification (Table 1). In CRC, low MAEA levels were also significantly associated with lymph node metastasis, distant metastasis, higher TNM stage, and higher CEA levels (Table 1). These data support a potential link between MAEA and the suppression of GC and CRC progression.

### MAEA suppresses the *in vitro* tumorgenesis of GIC cells

**Figure 2:**
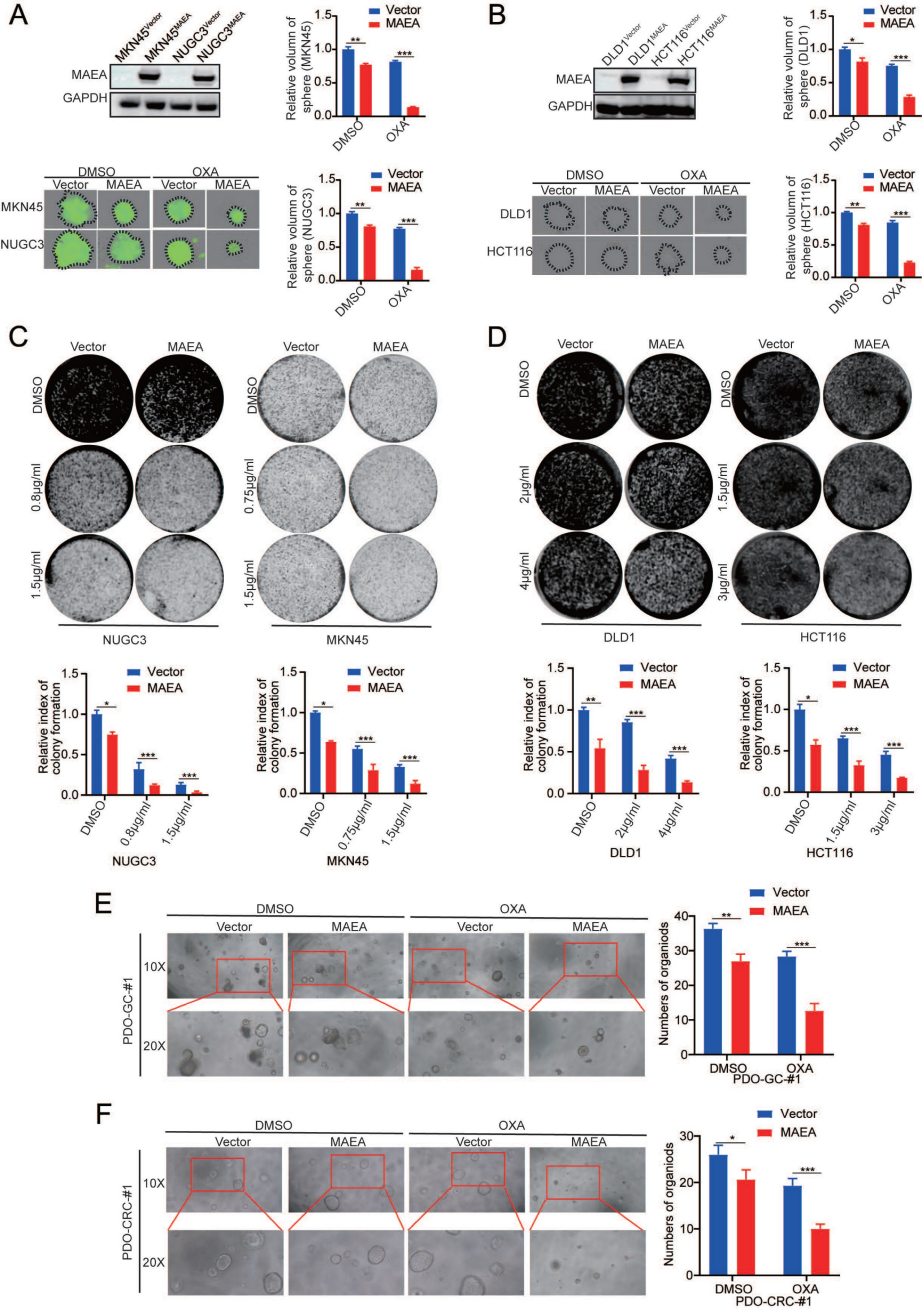
** MAEA suppresses the *in vitro* tumorgenesis of GIC cells. A-B** Representative images of sphere formation assays for the indicated cell types in the presence or absence of OXA. **C-D** Representative colony formation assay results for the indicated cell types in the presence or absence of OXA. **E-F** Representative organoid images for cells in which MAEA was overexpressed in the presence or absence of OXA treatment. Significance was as defined in prior figures.

The ability to form clones is one of the main ways to detect tumor cell occurrence. Lentiviral transduction was used to establish GC and CRC cell lines in which MAEA was stably overexpressed (Fig. 2A, B). Such MAEA overexpression significantly suppressed the sphere-forming and colony-forming abilities of these tumor cells in the presence or absence of oxaliplatin (Fig. 2A-D). Consistently, organoid formation experiments yielded comparable results (Fig. 2E, F). Thus, these results suggest that MAE can inhibit GIC tumorgenesis *in vitro*.

### MAEA suppresses the *in vitro* proliferation and oxaliplatin resistance of GIC cells

**Figure 3:**
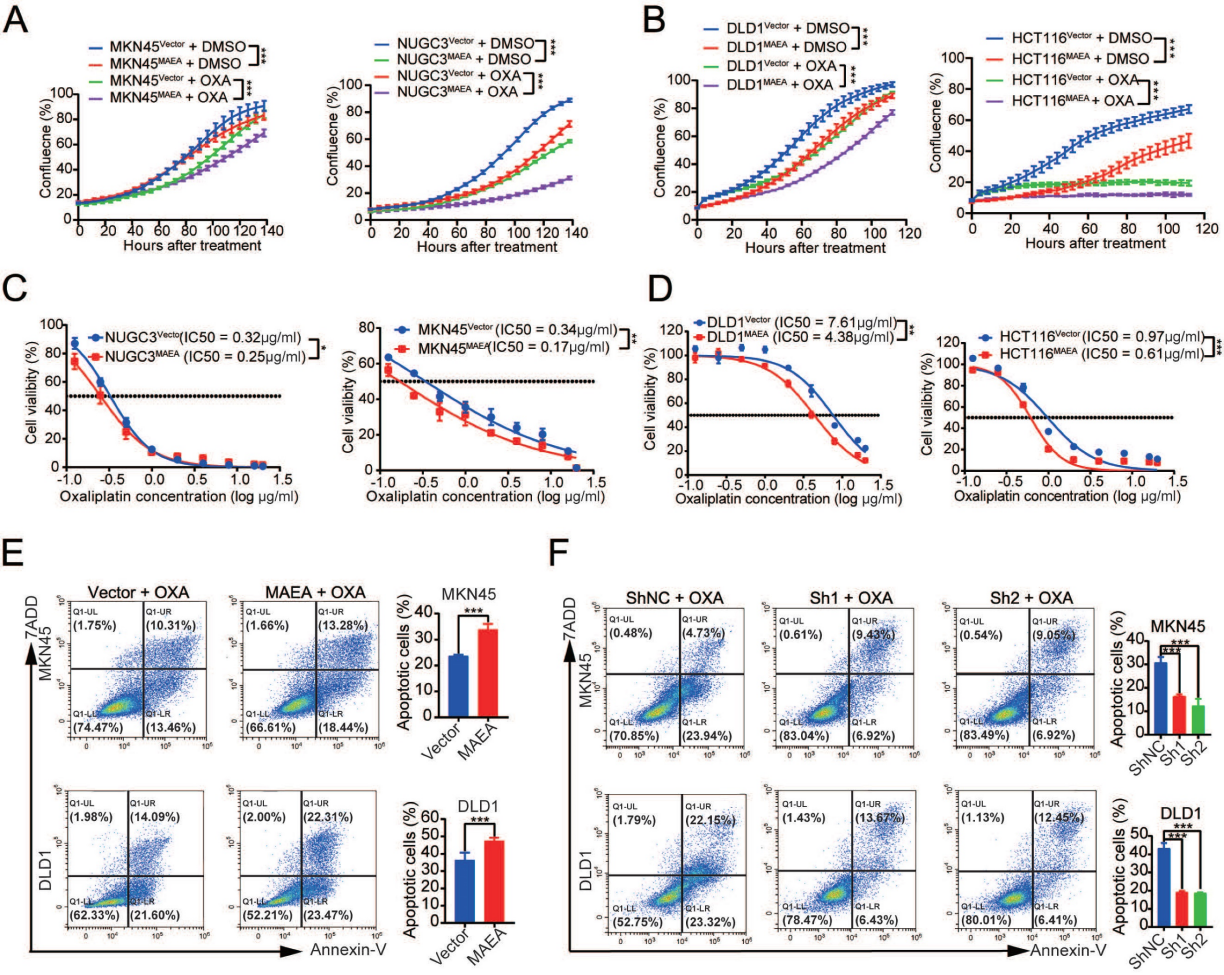
** MAEA suppresses the in vivo proliferation and oxaliplatin resistance of GIC cells. A-B** Growth curves for the indicated cell types under conditions of OXA treatment. **C-D** The survival of the indicated cell types was quantified following a 96 h treatment with a range of OXA concentrations. Significance was as defined in prior figures.** E-F** Apoptotic death for MAEA-overexpressing (E) or MAEA-knockdown (F) or control cells was assessed via flow cytometry following treatment for 48 h with OXA. Significance was as defined in prior figures.

Since MAEA can inhibit GIC tumorgenesis *in vitro*, we further verified the effect of MAEA on proliferation and drug resistance *in vitro*. MAEA overexpression also led to a significant drop in the proliferation of tumor cells that were or were not treated with oxaliplatin (Fig. 3A, B), in addition to significantly reducing the oxaliplatin IC50 value for these cell lines (Fig. 3C, D). We further investigated the effect of MAEA on GIC cell apoptosis using flow cytometry. Overexpression of MAEA significantly enhanced Oxaliplatin-induced apoptosis, whereas knocking down MAEA markedly decreased Oxaliplatin-induced apoptosis (Fig. 3E, F). MAEA is thus capable of suppressing GIC cell proliferation and oxaliplatin chemotherapy resistance *in vitro*.

### MAEA suppresses the *in vivo* proliferation and oxaliplatin resistance of GIC cells

**Figure 4:**
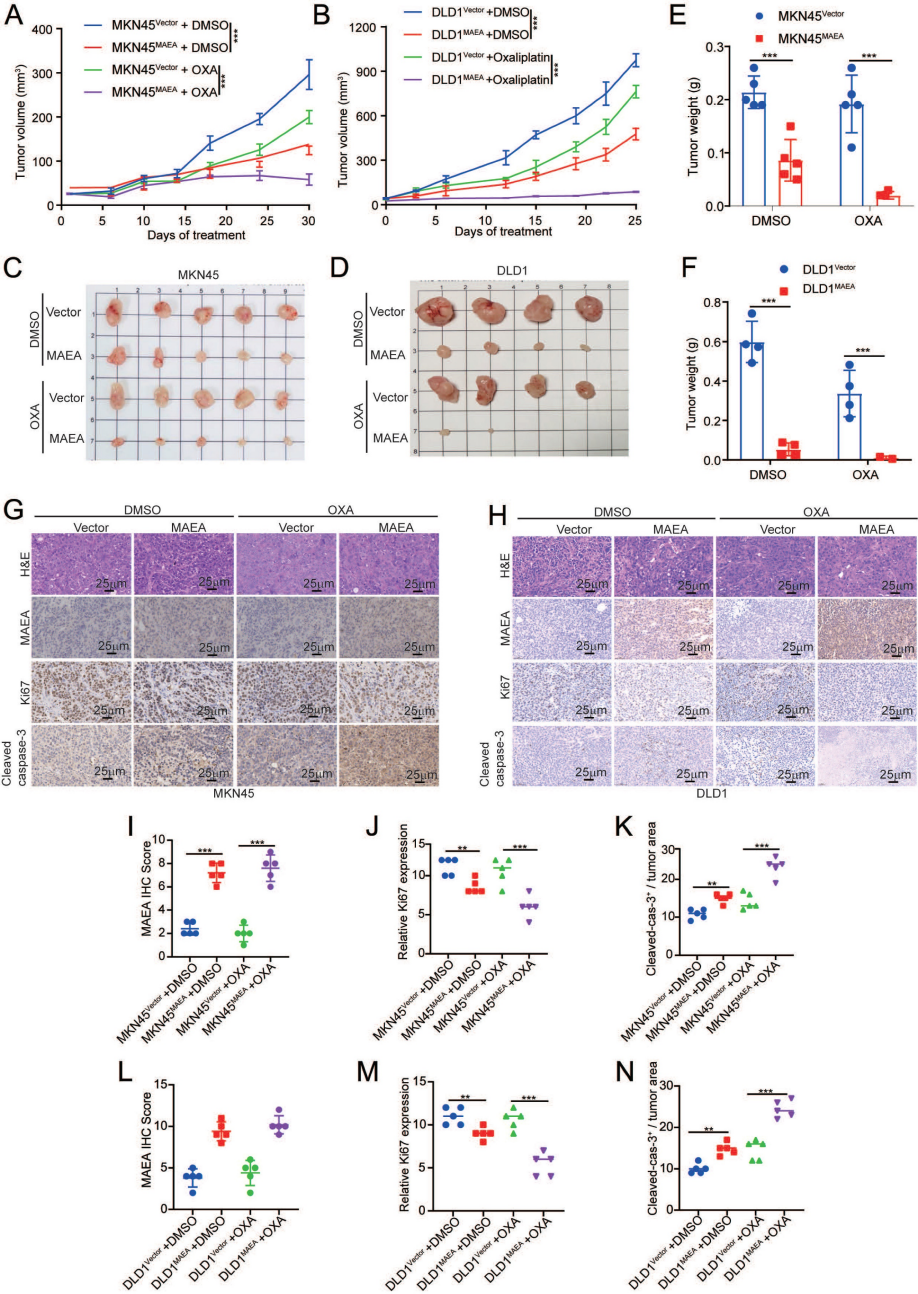
** MAEA suppresses the proliferation and oxaliplatin resistance of GI cancer cells *in vivo.* A-B** MKN45-Vector/MAEA and DLD1-Vector/MAEA tumors growth curves following transplantation in mice that were or were not subjected to OXA treatment (n = 5/group). **C-D** Tumor images following the defined treatment interval. **E-F** Tumor weights after the defined treatment interval. **G-H** Representative H&E, Ki67, and cleaved caspase-3 staining images for tumor sections from the indicated groups. **I-N** MAEA, Ki67 and cleaved caspase-3 staining results were quantified in the indicated groups through IHC staining. Significance was as defined in prior figures.

Subcutaneous GI tumor models were next established in nude mice using cell lines in which MAEA was stably overexpressed in order to assess the effects of this E3 ligase on tumor cell proliferation and oxaliplatin resistance *in vivo.* Relative to control tumors, both GC and CRC tumors in which MAEA was overexpressed exhibited impaired growth relative to corresponding controls, and this effect was even more pronounced in the context of oxaliplatin treatment (Fig. 4A-F). IHC staining revealed that MAEA significantly reduced Ki67 expression and promoted cleaved caspase-3 expression in GC and CRC tumor cells (Fig. 4G-L). These data thus suggest that MAEA can suppress the proliferation and oxaliplatin resistance of GIC cells *in vivo*.

### MAEA induces the ubiquitination and degradation of PARP1

**Figure 5:**
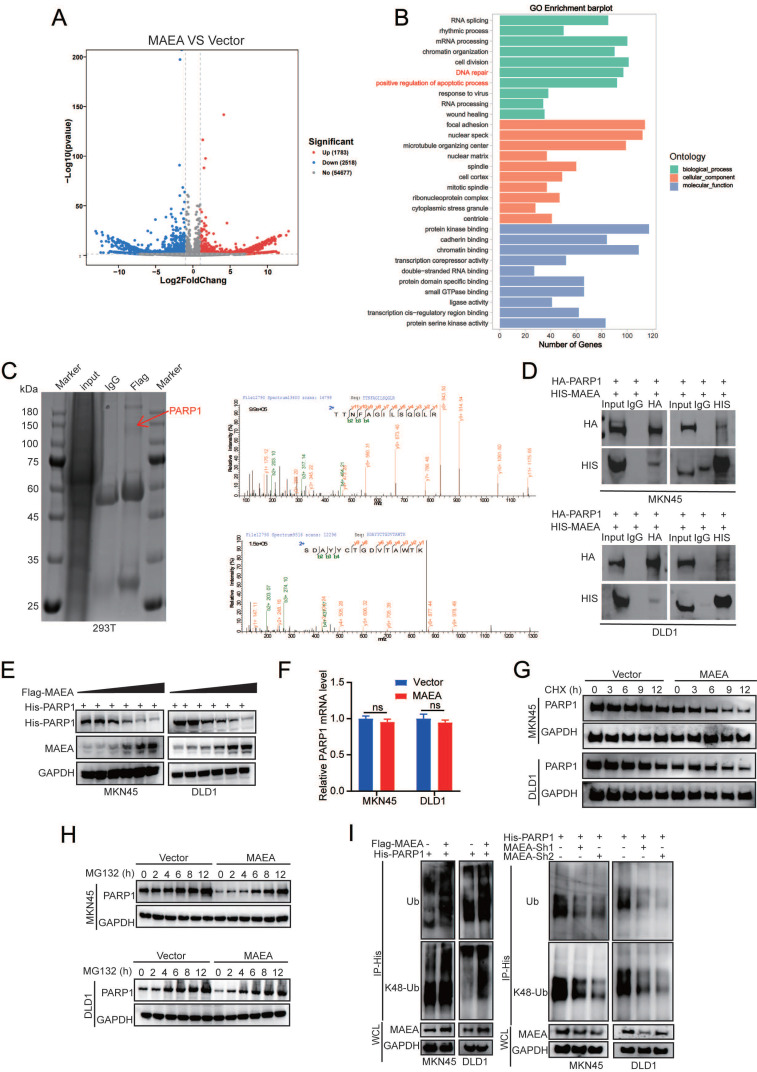
** MAEA induces the ubiquitination and degradation of PARP1. A** A volcano plot showing the genes that were differentially expressed relative to vector control following MAEA overexpression in MKN45 cells. **B** GO analyses were used to identify key biological processes associated with differentially expressed genes. **C** FLAG immunoprecipitation was performed in 293T cells in which Flag-MAEA was overexpressed, followed by the SDS-PAGE separation of these proteins, the manual excision of bands in the 75-150 kDa range, and their analysis via mass spectrometry. **D** Co-IP was performed with antibodies specific for HA and 6xHis, revealing that MAEA and PARP1 interact with one another. **E-F** MAEA reduced PARP1 protein but not mRNA levels in a dose-dependent fashion. **G-H** Western blotting was used to detect the levels of PARP1 levels in MKN45 and DLD1 cells transfected with MAEA that had been treated with CHX and MG132.** I** PARP1 ubiquitination in MKN45 and DLD1 cells was detected via Western immunoblotting in cells transfected with the indicated plasmids. Significance was as defined in prior figures.

To gain additional insight into the mechanistic basis for the suppression of GIC cell proliferation and oxaliplatin resistance by MAEA, MKN45-Vector/MAEA cells were analyzed via RNA sequencing. This approach revealed that MAEA overexpression was associated with the upregulation of 1,783 genes and the downregulation of 2,518 genes (Fig. 5A). Gene Ontology (GO) analyses of the genes differentially expressed when comparing these two tumor types revealed that MAEA is most closely associated with DNA repair and the positive regulation of apoptotic signaling (Fig. 5B). Immunoprecipitation and mass spectrometry were further used to identify MAEA-interacting proteins (Fig. 5C). Of the several MAEA-interacting targets identified with this approach, PARP1 was among the most significant, and the ability of the two to interact with one another was verified in both MKN45 and DLD1 cells (Fig. 5D). MAEA was capable of reducing PARP1 protein levels (Fig. 5E) without any corresponding effect at the mRNA level (Fig. 5F), suggesting that MAEA is a post-transcriptional PARP1 regulator. Relative to control cells, MAEA overexpression resulted in the significant acceleration of PARP1 degradation in cycloheximide (CHX)-treated cells (Fig. 5G). This MAEA-induced decrease in PARP1 protein levels was also mitigated by the proteasome inhibitor MG132 (Fig. 5H), suggesting that the ubiquitin-proteasome pathway may be responsible for the degradation of PARP1 in this system. Consistently, MAEA overexpression led to an increase in PARP1 K48-linked polyubiquitination that resulted in a drop in PARP1 protein levels (Fig. 5I). MAEA thus appears to promote PARP1 degradation by targeting it for K48-linked ubiquitination.

### MAEA inhibits the M2 polarization of macrophages while enhancing macrophage phagocytic activity

**Figure 6:**
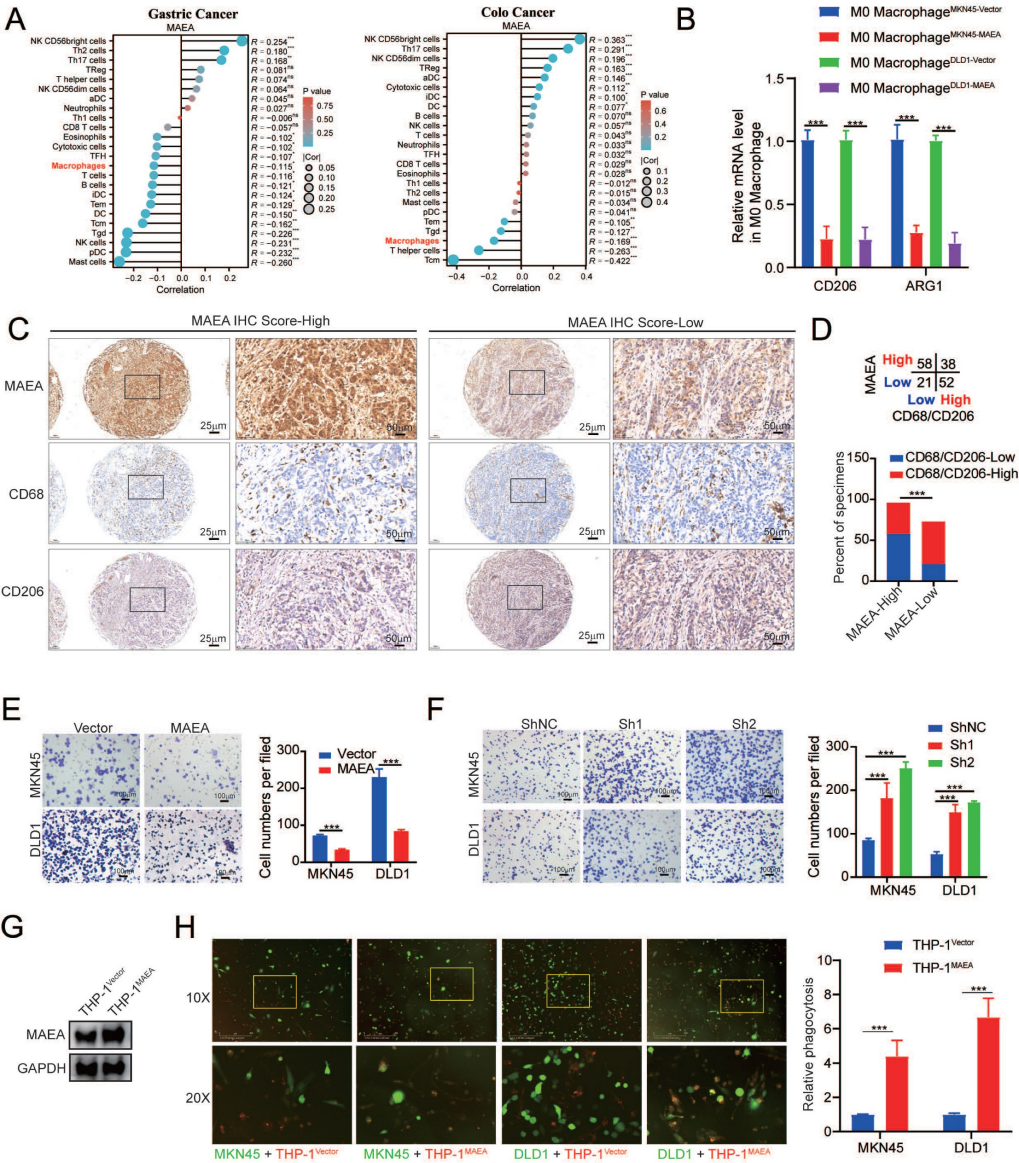
** MAEA inhibits the M2 polarization of macrophages while enhancing macrophage phagocytic activity. A** Analysis of data from the TCGA database revealed that MAEA expression and macrophage infiltration are significantly negatively correlated in GC and CRC. **B** qPCR analyses indicated that overexpressing MAEA led to significant decreases in CD206 and ARG1 expression in M0 macrophages when co-cultured with MAEA-overexpressing MKN45 and DLD1 cells. **C-D** Representative IHC staining results for MAEA, CD68, and CD206 in GC tumor tissues, revealing that MAEA expression is negatively correlated with CD68/CD206 protein levels. **E-F** Macrophages were cultured in the presence of cells in which MAEA was overexpressed or knocked down, and their migratory activity was analyzed. **G-H** THP1 cells were transfected to overexpress MAEA, differentiated into macrophages, and their ability to phagocytose tumor cells was analyzed. Significance was as defined in prior figures.

While it was named based on its characteristics as a macrophage erythroblast attacher, no research to date has examined the association between MAEA and macrophages in GIC. In the TCGA database, MAEA expression was significantly negatively correlated with macrophage infiltration in GC and CRC (Fig. 6A). MAEA overexpression was also associated with significant decreases in the mRNA expression levels of CD206 and ARG1, which are M2 macrophage markers (Fig. 6B), when co-cultured with MAEA-overexpressing MKN45 and DLD1 cells. This indicates that MAEA overexpression significantly inhibits the M2 polarization of macrophages. IHC staining results from 169 GC cases further demonstrated a significant negative correlation between MAEA levels and M2 (CD68/CD206) macrophage infiltration within tumor tissue (Fig. 6C, D). In a Transwell-based co-culture assay, MAEA was able to significantly suppress macrophage migration (Fig. 6E, F). When THP1 cells overexpressing MAEA were differentiated into macrophages, elevated levels of this E3 ligase were found to promote significantly enhanced macrophage-mediated tumor cell phagocytosis (Fig. 6G, H). As such, MAEA appears to suppress the M2 polarization of macrophages while enhancing macrophage phagocytosis, ultimately counteracting GIC progression.

### PARP1 inhibition in combination with MAEA overexpression enhances GIC cell sensitivity to oxaliplatin

**Figure 7:**
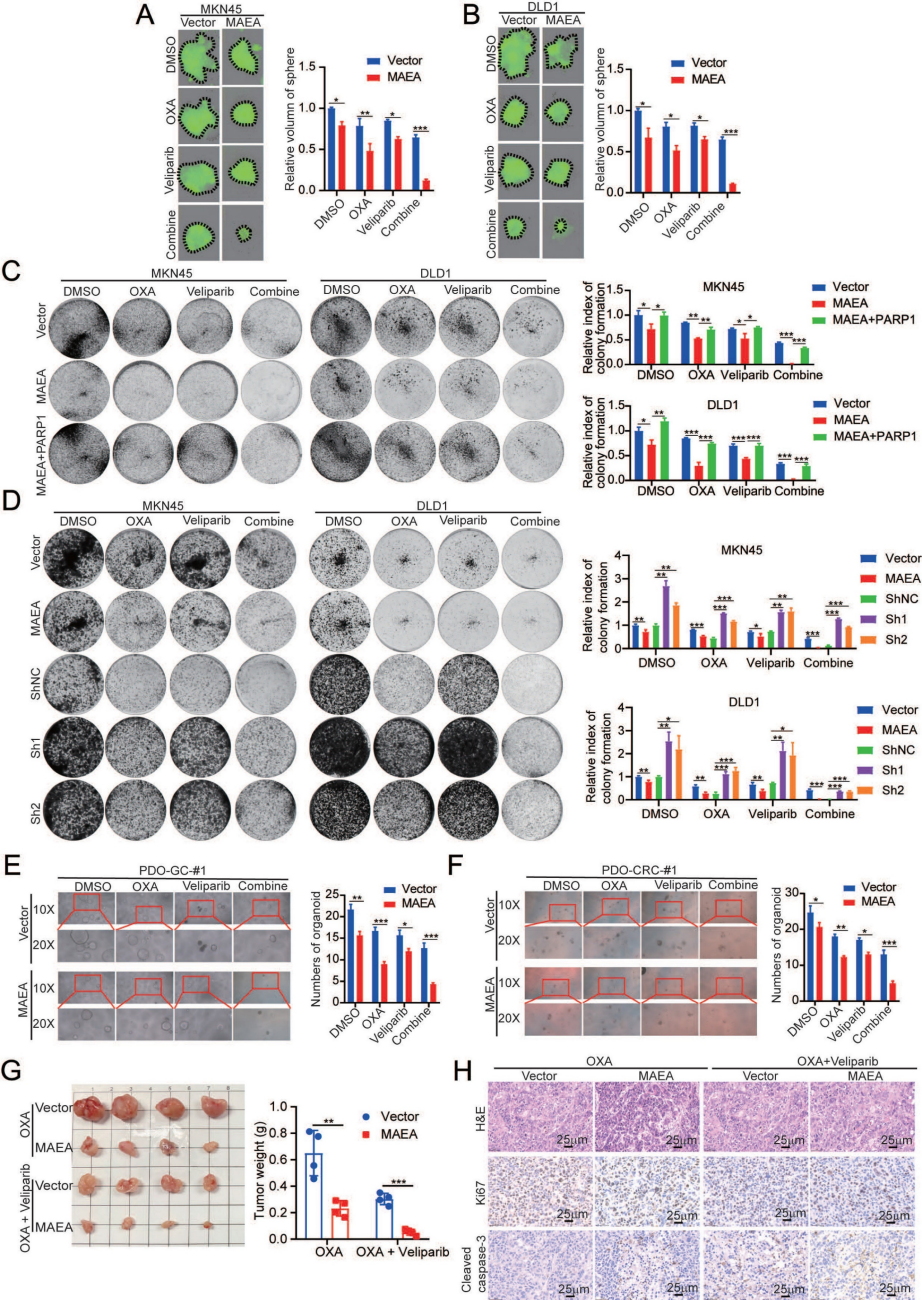
** PARP1 inhibition enhances the efficacy of oxalipatin treatment in the context of MAEA overexpression. A-B** Representative sphere formation assay results for MKN45 and DLD1 cells transfected with MAEA and subjected to OXA and/or Veliparib treatment. **C-D** Representative colony formation assay results for cells treated as in (A). **E-F** MAEA overexpression or empty control vectors were transfected into patient-derived organoids which were then treated using Veliparib and oxaliplatin and monitored for growth. **G** Xenograft tumor size and weight were analyzed for xenografts transfected to overexpress MAEA and treated with OXA alone or in combination with Veliparib. **H** H&E, Ki67, and cleaved caspase-3 staining were performed for the tumors from (G). Significance was as defined in prior figures.

Next, experiments were performed with the aim of verifying the preclinical utility of MAEA overexpression and Veliparib-mediated PARP1 inhibition. Treatment with Veliparib led to the significant enhancement of the ability of oxaliplatin to suppress sphere formation for MKN45 and DLD1 cells in which MAEA was overexpressed (Fig. 7A, B). PARP1 overexpression, in contrast, was able to overcome the inhibition of GIC clonogenesis mediated by MAEA, whereas Veliparib treatment had the opposite effect (Fig. 7C, D). Similar effects were also evident in organoid models of GC and CRC (Fig. 7E, F). These data suggest that Veliparib-mediated PARP1 inhibition can enhance oxaliplatin-mediated suppression of tumor cell growth for these cancer types. In mice, the combination of Valiparib and oxaliplatin also led to the significant suppression of MAEA-overexpressing subcutaneous tumor growth (Fig. 7G) with concomitant changes in Ki67 and cleaved caspase-3 (Fig. 7H) staining in tumors from these animals. The synergistic effects of MAEA and PARP1 inhibitor treatment can thus significantly enhance CRC and GC cell sensitivity to oxaliplatin treatment.

### MAEA overexpression and PARP1 inhibition enhance oxaliplatin sensitivity *in vivo*

**Figure 8:**
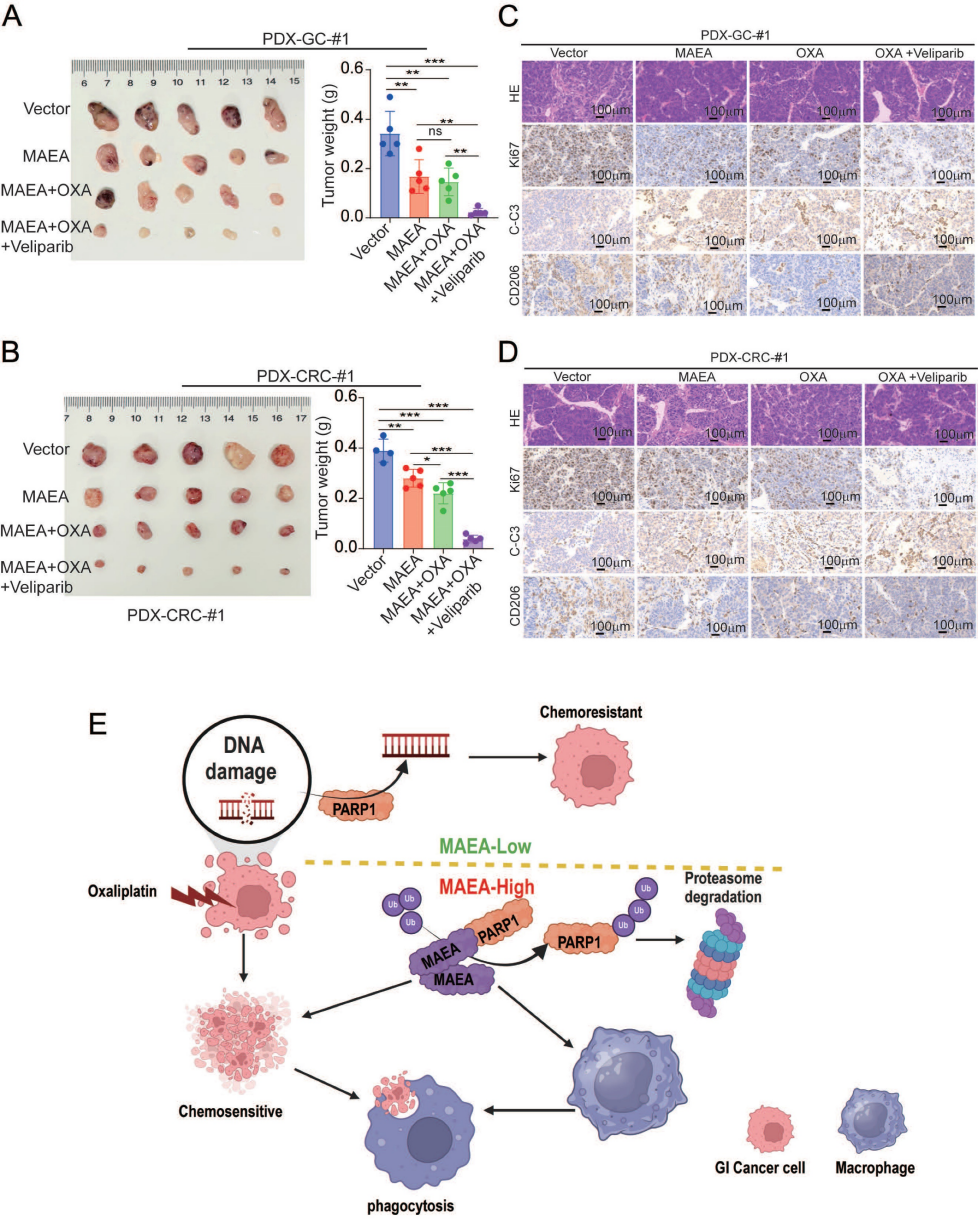
** MAEA overexpression and PARP1 inhibitor treatment enhance the *in vivo* preclinical efficacy of oxaliplatin treatment. A-B** Tumor weights were measured in the indicated treatment groups.** C-D** H&E, Ki67, and Cleaved caspase-3 staining were performed in the indicated treatment groups. **E** A schematic overview of the proposed mechanisms through which MAEA regulates malignant gastrointestinal tumor progression (Generated with BioRender). Significance was as defined in prior figures.

In a final series of preclinical experiments, the therapeutic effects of combining MAEA overexpression and Veliparib as a means of achieving greater oxaliplatin sensitivity were examined using patient-derived xenograft (PDX) models of GC and CRC. In these analyses, MAEA lentiviral transduction markedly hampered tumor growth relative to control tumors, while combined Veliparib treatment resulted in profoundly enhanced oxaliplatin therapeutic efficacy characterized by the near total suppression of PDX tumor growth (Fig. 8A, B). Immunohistochemical staining further revealed that MAEA strongly suppressed the expression of the proliferation marker Ki67 and the M2 macrophage marker CD206 in these PDX tumors while increasing the levels of intratumoral apoptosis-associated cleaved caspase-3 (Fig. 8C, D). These results indicate that patients with high MAEA expression may achieve better preclinical treatment outcomes using OXA combined with Veliparib.

## Discussion

While oxaliplatin-based chemotherapy remains a first-line approach to the management of GC and CRC, the emergence of oxaliplatin resistance remains a persistent barrier to therapeutic efficacy. There is thus a clear need to clarify the mechanisms underlying drug resistance and to identify key related targets for efforts to improve outcomes for patients with these forms of cancer 23, 24. Here, the functions of the E3 ubiquitin ligase MAEA were explored for the first time in GIC, highlighting its relevance as a therapeutic target. Specifically, MAEA was found to be significantly linked with patient outcomes such that higher MAEA expression levels were linked to better OS and DFS. In contrast, prior research conducted in glioblastoma has revealed that MAEA can ubiquitinate and degrade PHD3, thereby promoting disease progression 18, while studies have also demonstrated that PHD3 inhibits the metastasis of colon cancer through the occludin-p38 pathway 25, reduces the migratory and invasive capacity of gastric cancer cells, and impedes the formation of tumor vasculature by negatively regulating HIF1A and VEGF 26. These findings suggest that MAEA might also be involved in the regulation of gastrointestinal cancers.

In contrast, MAEA suppressed proliferative and chemoresistance activity in GIC tumors in this study *in vitro* and *in vivo* while also enhancing the phagocytic capacity of macrophages. At the molecular level, these effects were mediated at least in part by interactions between MAEA and PARP1 that resulted in the K48-linked ubiquitination and proteasomal degradation of this DNA damage repair pathway protein. This activity, in turn, inhibits proliferation and renders GIC cells more sensitive to oxaliplatin. Notably, MAEA was able to attenuate the migration and M2 polarization of macrophages. Combining MAEA and the PARP1 inhibitor Veliparib also led to a significantly better oxaliplatin response in preclinical mouse model systems.

Chemotherapy causes extensive DNA damage in tumor cells 11, 27, but these malignant cells can engage DNA damage repair pathways to achieve chemoresistance, with PARP1-mediated DNA damage response activity being particularly important in this context 28. PARP1 plays a role in single-strand break repair and base excision repair, with its hyperactivity or overexpression contributing to more efficient DNA repair and consequent chemoresistance 29. The inhibition of PARP1, in contrast, can render tumor cells more sensitive to chemotherapy as a result of the prevention of DNA damage repair, resulting in the enhanced cytotoxic efficacy of chemotherapeutic drugs 30. Here, MAEA was found for the first time to promote PARP1 ubiquitination and degradation, in turn suppressing tumor cell proliferation and chemoresistance.

Veliparib is a PARP1 inhibitor that can sensitize tumor cells to chemotherapeutic and radiotherapeutic treatment owing to the disruption of DNA damage repair processes 31, 32. The efficacy of Veliparib alone or in combination with various chemotherapeutic drugs has been documented efficacy in ovarian, breast, lung, and prostate cancer 33. In clinical trials, it has been shown to enhance chemotherapeutic efficacy, prolong progression-free survival, and help overcome resistance to some forms of treatment 13, 14. While studies of Veliparib in GC have been limited, the present results underscore its potential beneficial effects in this context. Specifically, Veliparib was able to significantly enhance the effects of oxaliplatin treatment following the overexpression of MAEA. Consistent results were observed across GC and CRC preclinical model systems, suggesting that Veliparib may be a valuable adjuvant treatment for these forms of GIC. Additional clinical trials, however, will be necessary to thoroughly evaluate the safety and efficacy of Veliparib in these indications.

Macrophage phagocytic activity can have a strong impact on chemotherapeutic efficacy 34, 35. Through their ability to enhance apoptotic tumor cell clearance, macrophages can clear residual tumor cells and facilitate regression after chemotherapeutic treatment. In this study, the previously unrecognized ability of MAEA to promote phagocytic activity in macrophages was identified. MAEA was able to enhance apoptotic death in response to oxaliplatin through the inhibition of PARP1 while also simultaneously enhancing macrophage-mediated phagocytic activity. Future studies, however, will be necessary to clarify the specific mechanisms through which MAEA can enhance macrophage phagocytosis. Due to budgetary constraints, we were unable to collect tissue samples from patients for further single-cell analysis of MAEA expression. However, to address this aspect, we performed a comprehensive single-cell analysis using the publicly available TISCH (Tumor Immune Single-cell Hub, http://tisch.comp-genomics.org/home/) database. Our analysis revealed that MAEA is significantly expressed in macrophages within the tumor microenvironment of colorectal cancer tissues (Figure S1A). Moreover, the single-cell analysis also indicated that MAEA expression is negatively correlated with prognosis in gastrointestinal cancers (Figure S1B). Importantly, its expression in immune cells, particularly macrophages, showed a remarkable specificity, underscoring its potential role in modulating the tumor-immune microenvironment (Figure S1C-E). These findings provide valuable insights into the expression patterns of MAEA and suggest distinct roles for MAEA in macrophages compared to tumor cells. While MAEA in macrophages might be involved in immune regulation and shaping the tumor microenvironment, its expression in tumor cells may be more directly linked to oncogenic pathways or tumor progression.

## Conclusions

The results of this study suggest that MAEA can induce PARP1 ubiquitination and degradation while simultaneously augmenting macrophage phagocytic activity. Through these complementary mechanisms, MAEA can suppress the proliferation of tumor cells and mitigate their resistance to oxaliplatin. MAEA thus holds promise as a diagnostic biomarker and therapeutic target when seeking to overcome oxaliplatin chemoresistance during the treatment of GIC.

## Supplementary Material

Supplementary figures and tables.

## Figures and Tables

**Table 1:** Correlations between the expression of MAEA and clinicopathological characteristics of GC and CRC patients.

Clinicopathological Characteristics (GC)	Low MAEA	High MAEA	*p-* Value	Clinicopathological Characteristics (CRC)	Low MAEA	High MAEA	*p-* Value
Number	73	96		Number	322	322	
Age, n (%)			0.193	Age, n (%)			0.265
<60 years	37 (50.7)	39 (36.4)		<= 65	145 (45)	131 (40.7)	
≥60 years	36 (49.3)	57 (63.6)		> 65	177 (55)	191 (59.3)	
Gender, n (%)			0.154	Gender, n (%)			0.477
Male	56 (76.7)	64 (74.5)		Male	167 (51.9)	176 (54.7)	
Female	17 (23.3)	32 (25.5)		Female	155 (48.1)	146 (45.3)	
Invasion Depth, n (%)			0.153	Invasion Depth, n (%)			0.159
T1 + T2	11 (15.1)	23 (23.9)		T1 + T2	58 (18.2)	73 (22.7)	
T3 + T4	62 (84.9)	73 (76.1)		T3 + T4	261 (81.8)	249 (77.3)	
Lymph Node Metastasis, n (%)			0.065	Lymph Node Metastasis, n (%)			0.007
N0	13 (17.8)	29 (30.2)		N0	166 (52.2)	202 (62.7)	
N1/N2/N3	60 (82.2)	67 (69.8)		N1&N2	152 (47.8)	120 (37.3)	
Distant Metastasis, n (%)			0.014	Distant Metastasis, n (%)			0.020
M0	53 (72.6)	84 (87.5)		M0	219 (80.5)	256 (87.7)	
M1	20 (27.4)	12 (12.5)		M1	53 (19.5)	36 (12.3)	
TNM Stage, n (%)			0.076	TNM Stage, n (%)			0.018
I+II	18 (24.7)	36 (37.5)		I+II	159 (51.3)	190 (60.7)	
III+IV	55 (75.3)	60 (62.5)		III+IV	151 (48.7)	123 (39.3)	
Perineural Invasion, n (%)			0.001	Perineural invasion, n (%)			0.359
Absent	21 (28.8)	58 (60.4)		No	99 (72.3)	76 (77.6)	
Present	52 (71.2)	38 (39.6)		Yes	38 (27.7)	22 (22.4)	
Vessel Invasion, n (%)			0.640	Lymphatic invasion, n (%)			0.155
Absent	32 (43.8)	67 (69.8)		No	181 (63.1)	169 (57.3)	
Present	41 (56.2)	29 (30.2)		Yes	106 (36.9)	126 (42.7)	
Differentiation, n (%)			0.231	CEA level, n (%)			0.019
Well-Moderately	59 (26.5)	70 (18.2)		<= 5	118 (57.3)	143 (68.4)	
Poor	14 (73.5)	26 (81.8)		> 5	88 (42.7	66 (31.6)	
Lauren's classification, n (%)			0.028	Residual tumor, n (%)			0.401
Diffuse type	35 (47.9)	40 (32.7)		R0	221 (92.9)	247 (90.8)	
Intestinal type	23 (31.5)	47 (50.9)		R1&R2	17 (7.1)	25 (9.2)	
Mixed type	15 (20.6)	8 (16.4)					
Histologic type, n (%)			0.133	Histological type, n (%)			0.943
Tubular or papillary adenocarcinoma	66 (90.4)	76 (85.5)		Adenocarcinoma	276 (86.8%)	274 (87%)	
Signet-ring cell carcinoma	6 (8.2)	14 (7.3)		Mucinous adenocarcinoma	42 (13.2%)	41 (13%)	
Mucinous adenocarcinoma	1 (1.4)	5 (5.5)					
^a^ Others	0 (0)	1 (1.7)					

Statistical analyses were performed with the Pearson χ^2^ test ^a^Other: hepatoid adenocarcinoma and squamous carcinoma

## Abbreviations

MAEA: macrophage erythroblast attacher
GIC: gastrointestinal cancer
GC: gastric cancer
CRC: colorectal cancer
PARP1: Poly ADP-ribose Polymerase 1
IHC: immunohistochemical
PDX: patient-derived xenograft
OS: overall survival
DFS: disease-free survival
GO: Gene Ontology
TCGA: The Cancer Genome Atlas
