# Supplementary material for: The E3 ubiquitin ligase MAEA promotes macrophage phagocytosis and inhibits gastrointestinal cancer progression by mediating PARP1 ubiquitination and degradation
Source: Int J Biol Sci. 2025 Feb 10;21(4):1784–800. doi: 10.7150/ijbs.102796 (PMC11844278; doi:10.7150/ijbs.102796)
Supplement: Supplementary file 1 — Supplementary figures and tables. [file ijbsv21p1784s1.pdf]

Uncropped Western blots

Figure 2A

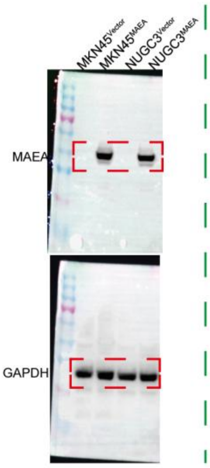

Figure 2B

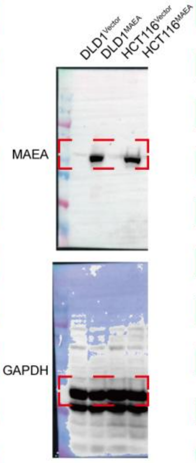

Figure 4D

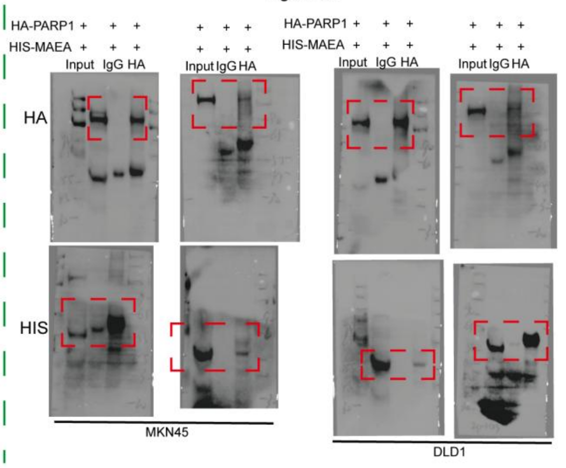

Figure 4E

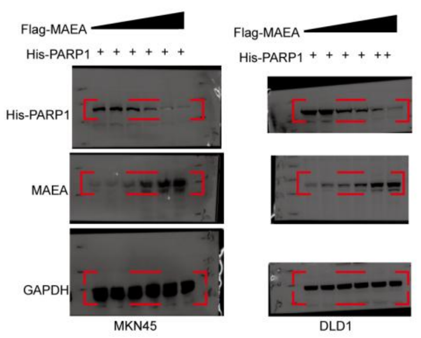

Figure 4G

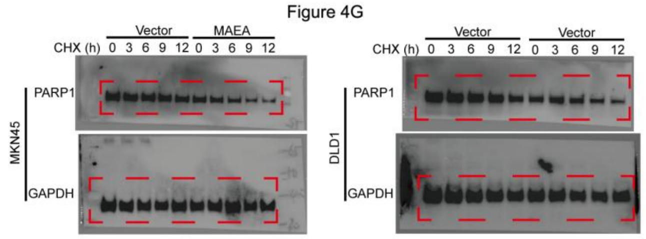

Figure 4H

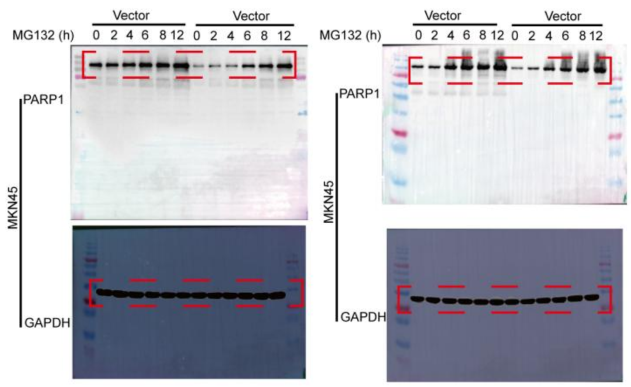

Figure 4I

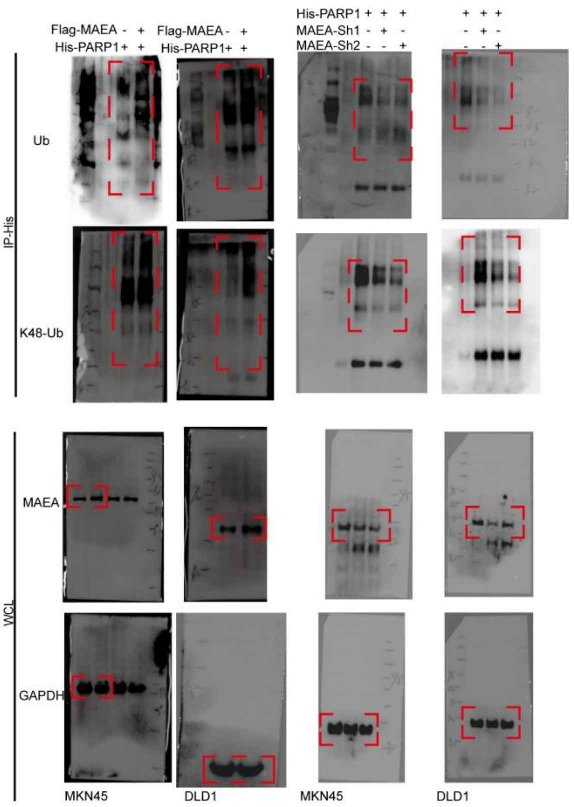

Figure S1

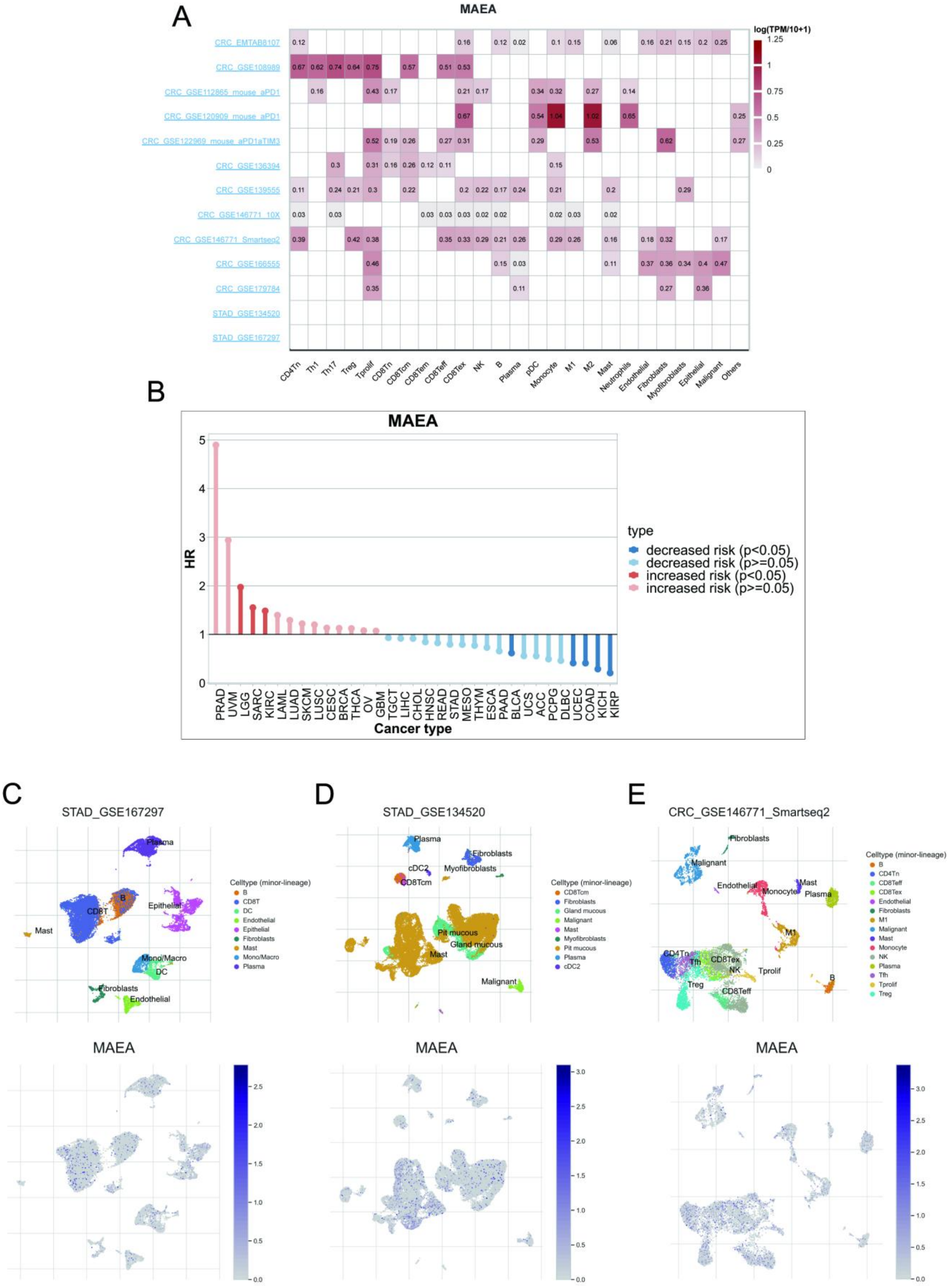

A
MAEA
log(TPM/10+1)
CRC_EMTAB8107
CRC_GSE108989
CRC_GSE112865_mouse_aPD1
CRC_GSE120909_mouse_aPD1
CRC_GSE122969_mouse_aPD1aTIM3
CRC_GSE136394
CRC_GSE139555
CRC_GSE146771_10X
CRC_GSE146771_Smartseq2
CRC_GSE166555
CRC_GSE179784
STAD_GSE134520
STAD_GSE167297
CD4Tn Th1 Th17 Treg Tprolif CD8Tn CD8Tcm CD8Tem CD8Teff CD8Tex NK B Plasma pDC Monocyte M1 M2 Mast Neutrophils Endothelial Fibroblasts Myofibroblasts Epithelial Malignant Others
B
MAEA
HR
type
decreased risk (p<0.05)
decreased risk (p>=0.05)
increased risk (p<0.05)
increased risk (p>=0.05)
PRAD UVM LGG SARC KIRC LAML LUAD SKCM LUSC CESC BRCA THCA OV GBM TGCT LIHC CHOL HNSC READ STAD MESO THYM ESCA PAAD BLCA UCS ACC PCPG DLBC UCEC COAD KICH KIRP
Cancer type
C
STAD_GSE167297
Celltype (minor-lineage)
MAEA
D
STAD_GSE134520
Celltype (minor-lineage)
MAEA
E
CRC_GSE146771_Smartseq2
Celltype (minor-lineage)
MAEA

Supplementary Table 1. *Correlation between MAEA expression and clinicopathological characteristics of gastric patients.*

Clinicopathological Characteristics	Low MAEA	High MAEA	*p*-Value
Number	73	96	
**Age, n (%)**			0.193
<60 years	37 (50.7)	39 (36.4)	
≥60 years	36 (49.3)	57 (63.6)	
**Gender, n (%)**			0.154
Male	56 (76.7)	64 (74.5)	
Female	17 (23.3)	32 (25.5)	
**Histologic type, n (%)**			0.133
Tubular or papillary adenocarcinoma	66 (90.4)	76 (85.5)	
Signet-ring cell carcinoma	6 (8.2)	14 (7.3)	
Mucinous adenocarcinoma	1 (1.4)	5 (5.5)	
[a] Others	0 (0)	1 (1.7)	
**Differentiation, n (%)**			0.231
Well-Moderately	59 (26.5)	70 (18.2)	
Poor	14 (73.5)	26 (81.8)	
**Lauren's classification, n (%)**			0.028
Diffuse type	35 (47.9)	40 (32.7)	
Intestinal type	23 (31.5)	47 (50.9)	
Mixed type	15 (20.6)	8 (16.4)	
**Invasion Depth, n (%)**			0.153
T1 + T2	11 (15.1)	23 (23.9)	
T3 + T4	62 (84.9)	73 (76.1)	
**Lymph Node Metastasis, n (%)**			0.065
N0	13 (17.8)	29 (30.2)	
N1/N2/N3	60 (82.2)	67 (69.8)	
**Distant Metastasis, n (%)**			0.014
M0	53 (72.6)	84 (87.5)	
M1	20 (27.4)	12 (12.5)	
**TNM Stage, n (%)**			0.076
I+II	18 (24.7)	36 (37.5)	
III+IV	55 (75.3)	60 (62.5)	
**Perineural Invasion, n (%)**			0.001
Absent	21 (28.8)	58 (60.4)	
Present	52 (71.2)	38 (39.6)	
**Vessel Invasion, n (%)**			0.640
Absent	32 (43.8)	67 (69.8)	
Present	41 (56.2)	29 (30.2)	

Statistical analyses were performed by the Pearson χ^2 test

[a]Others: hepatoid adenocarcinoma and squamous carcinoma

Supplementary Table 2. *Univariate and multivariate analyses for the prognostic factors of overall survival in gastric cancer patients.*

Variable	Univariate Analysis		Multivariate Analysis	
	**HR (95% CI)**	***p*-Value**	**HR (95% CI)**	***p*-Value**
Age (≥60 years vs. <60 years)	0.782 (0.506–1.210)	0.269	0.523 (0.302–0.907）	0.021
Gender (male vs. female)	1.348 (0.857–2.120)	0.196	1.396 (0.770–2.530)	0.272
Histology (tubular/papillary adenocarcinoma	1.763 (0.850–3.656)	0.128	1.492 (0.623–3.571)	0.369
vs. the others)				
Differentiation (well/moderate vs. poor)	0.657 (0.381–1.136)	0.133	0.584 (0.285–1.197)	0.142
Invasion depth (T3/T4 vs. T1/T2)	6.147 (2.484–15.215)	< 0.001	2.826 (0.907–8.804)	0.073
Lymph Node Metastasis (N+ vs. N0)	4.974 (2.393–10.339)	<0.001	2.686 (1.115–6.475)	0.028
Distant metastasis (M1 vs. M0)	3.553 (2.206–5.723)	<0.001	2.127 (1.182–3.826)	0.012
Perineural Invasion (present vs. absent)	3.323 (2.020–5.467)	<0.001	1.885 (0.916–3.878)	0.085
Vessel Invasion (present vs. absent)	2.125 (1.374–3.286)	0.001	1.136 (0.651–1.982)	0.653
MAEA expression (high vs. low)	0.211 (0.120–0.370)	<0.001	0.238 (0.128–0.446)	0.001

Supplementary Table 3. *Univariate and multivariate analyses for the prognostic factors of disease free survival in gastric cancer patients.*

**Variable**	**Univariate Analysis**		**Multivariate Analysis**	
	**HR (95% CI)**	***p*-Value**	**HR (95% CI)**	***p*-Value**
Age (≥60 years vs. <60 years)	0.782 (0.506–1.210)	0.269	0.814 (0.402-1.647)	0.556
Gender (male vs. female)	1.348 (0.857–2.120)	0.196	1.311 (0.624-2.754)	0.475
Histology (tubular/papillary adenocarcinoma	1.763 (0.850–3.656)	0.128	1.491 (0.587-3.789)	0.401
vs. the others)				
Differentiation (well/moderate vs. poor)	0.657 (0.381–1.136)	0.133	0.450 (0.179-1.128)	0.089
Invasion depth (T3/T4 vs. T1/T2)	6.147 (2.484–15.215)	< 0.001	2.847 (0.720-11.258)	0.136
Lymph Node Metastasis (N+ vs. N0)	4.974 (2.393–10.339)	<0.001	1.957 (0.723-5.295)	0.186
Distant metastasis (M1 vs. M0)	3.553 (2.206–5.723)	<0.001	1.006 (0.414-2.444)	0.989
Perineural Invasion (present vs. absent)	3.323 (2.020–5.467)	<0.001	1.753 (0.700-4.394)	0.231
Vessel Invasion (present vs. absent)	2.125 (1.374–3.286)	0.001	1.911 (0.964-4.110)	0.063
MAEA expression (high vs. low)	2.745 (1.625–4.638)	<0.001	0.327 (0.155-0.691)	0.003
